# Post-menopausal vaginal bleeding caused by carcinoma of the appendix: a case report

**DOI:** 10.1186/1752-1947-4-127

**Published:** 2010-05-02

**Authors:** Hans van Huisseling, Lennie van Hanegem, Martin van Dijk

**Affiliations:** 1 Department of Obstetrics and Gynecology, Groene Hart Ziekenhuis, PO Box 1098, 2800 BB Gouda, the Netherlands; 2 Department of Pathology, Groene Hart Ziekenhuis, PO Box 1098, 2800 BB Gouda, the Netherlands

## Abstract

**Introduction:**

Post-menopausal blood loss is a common complaint of patients seen in gynecological practice. The most frequent malignancy found in cases of post-menopausal bleeding is endometrial cancer. Other causes can be malignancies of the rest of a woman's genital tract or metastases from other tumors. To the best of our knowledge, it appears that this is the first published case of a post-menopausal primary appendiceal carcinoma presenting with vaginal blood loss.

**Case presentation:**

A 75-year-old Caucasian woman with a history of vaginal hysterectomy presented with a 10-month history of post-menopausal blood loss. After extensive examination and discussion, ovarian carcinoma was suggested. Microscopic examination of the tissue removed at laparotomy revealed an adenocarcinoma of the appendix. She was treated with adjuvant radiotherapy and with palliative chemotherapy after 14 months because of intra-abdominal metastatic disease.

**Conclusion:**

Post-menopausal blood loss in a patient with a history of hysterectomy is uncommon and always needs further investigation.

## Introduction

Post-menopausal blood loss is a common complaint of patients seen in gynecological practice. The most frequent malignancy found in cases of post-menopausal bleeding is endometrial cancer. Other causes of malignant post-menopausal blood loss can be carcinomas of a woman's genital tract (vagina, cervix, fallopian tubes or ovaries) or metastases from other tumors [1,2]. Post-menopausal bleeding with a history of hysterectomy is rather uncommon. We present a case of post-menopausal blood loss in a hysterectomized patient caused by carcinoma of the appendix.

To the best of our knowledge, it appears that this is the first case of a post-menopausal primary appendiceal carcinoma presenting with vaginal blood loss.

## Case presentation

A 75-year-old Caucasian woman was referred to our hospital with a 10-month history of vaginal bleeding. In 1986, she underwent a hysterectomy because of dysfunctional uterine bleeding. The cause of the blood loss was initially interpreted as vaginal atrophy which was unsuccessfully treated with estriol cream. She had experienced several urinary tract infections, which she never had before. She did not have any other complaints.

On physical examination, it was found that there was no palpable abdominal mass. On vaginal examination, a crater-shaped lesion was found in the right upper part of the vagina, which indurated the surrounding tissue, with a fetid smell and necrosis. Rectal examination showed no abnormalities.

Transvaginal ultrasound showed a 30 × 22 mm tumor on the top of the vagina. No ascites were seen. A biopsy revealed an adenocarcinoma. Immunohistochemical staining was positive for cytokeratin 20 and carcinoembryonic antigen (CEA), and negative for cytokeratin 7 and carbohydrate antigen (CA)-125, suggesting the origin of the tumor was more likely to be gastrointestinal than urogenital.

Laboratory findings, including tumor markers, were all within normal values, except for CEA (Immulite 2500, Siemens Medical Solution Diagnostics, LA, USA), which was raised at 16 μg/L (normal 2-4 μg/L).

**Figure 1:**
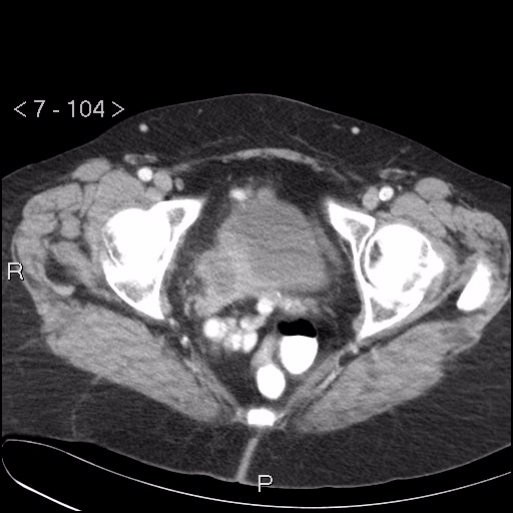
**Post-contrast computed tomography scan (Siemens Positron Plus 4) showing a tumorous mass located at the right adnexal region with a broad vaginal cuff bordering a thickened bladder wall**.

Pre-operative exams (chest X-ray, colonoscopy and cystoscopy) did not show any characteristic malignancy or metastasis. A computed tomography (CT) scan showed a process in the right ovary bed reaching the vaginal vault and medial side of the urinary bladder (Figure 1). It did not exclude bladder infiltration. Biopsies taken during cystoscopy showed extensive inflammation, but no signs of malignancy. Biopsies taken from the cecum showed adenomatous tissue with low-grade non-malignant dysplasia. The radiologist suggested a diagnosis of ovarian carcinoma. After discussion in our multidisciplinary oncology team, a laparotomy was decided upon in order to determine staging and/or plan cytoreductive surgery.

During laparotomy, it was observed that the vaginal vault was infiltrated by an enlarged tumorous appendix, with two loops of the ileum attached to the process. No infiltration in the bladder was seen. A right hemicolectomy was performed on part of the upper vagina. Both ovaries had a normal atrophic aspect.

**Figure 2:**
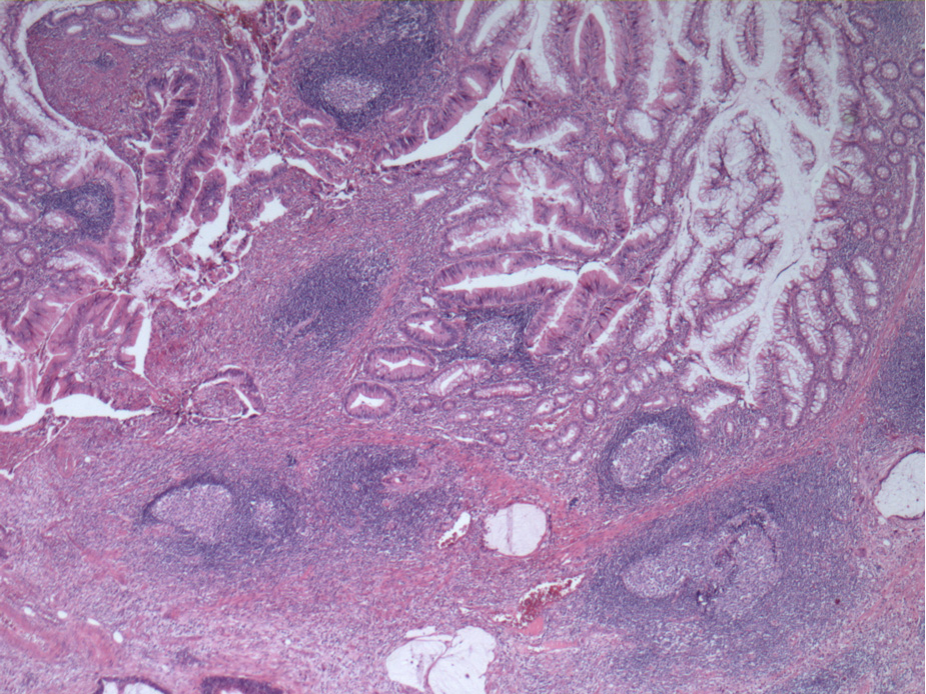
**Low power appearance of the colonic type appendiceal adenocarcinoma arising in a villous adenoma**.

Microscopic examination of the tissue showed a primary adenocarcinoma of the appendix, 1 cm in diameter, arising in a colonic type villous adenoma (Figure 2). There was extensive infiltration in the mesoappendix and ileum. One out of 14 dissected lymph glands showed a metastasis. Both mucosal cutting edges were free of tumor, but it extended into the vaginal cutting edge: pT4N1 M0. It was decided to give our patient adjuvant radiotherapy: she received 50.4Gy in 28 fractions of 1.8Gy on the vaginal vault and the original tumor location. She also received brachytherapy of 14Gy high-dose rate (HDR) in two fractions of 7Gy, 5 mm from the surface and 5 mm from the top with a one-week interval.

Fourteen months after surgery, during a transvaginal ultrasound in a regular follow-up, the tumor was found to have recurred. Ascites were also seen. Abdominal and pelvic CT scans revealed extensive intra-abdominal tumor spread with deposits on the diaphragm, omentum, vaginal vault and the sigmoid colon.

The multidisciplinary oncology team advised palliative treatment with the combination oxaliplatin-capecitabine, as the tumor was colon-like. After three cycles of chemotherapy, CA-125 levels decreased from 162 to 86 kU/L (Immulite 2500) and a CT scan showed significant reduction of the tumor deposits.

## Discussion

Post-menopausal vaginal bleeding is a common complaint of patients seen in gynecological practice. It accounts for approximately 5% of all gynecological visits [3]. Every case of post-menopausal bleeding is abnormal and should be investigated for any malignancy until proven otherwise [1,4]. The most frequent malignancy found in cases of post-menopausal bleeding is endometrial cancer. However, our patient had a hysterectomy in 1986. In our case, the vaginal examination was sufficient to suggest a malignant cause for the vaginal bleeding, because of the crater-shaped lesion found, and the indurated and necrotic tissue.

Primary carcinoma of the vagina is rare. It represents only 2% of all gynecological malignancies [5]. Most of these tumors are found in patients whose mothers used diethylstilbestrol (DES) during pregnancy. About 0.1% of prenatal exposed women develop vaginal carcinomas [6]. Since DES was prescribed to pregnant women from 1947 to 1976 in The Netherlands, our patient was too old to be a so-called 'DES daughter'.

The differential diagnosis included metastasis from an unknown primary tumor, carcinoma of the ovary and an intestinal tumor with infiltration in the vagina. Our patient had no complaints that suggested malignancy of the colon. Furthermore a colonoscopy showed no abnormalities. A recent mammogram was also normal. Only the raised level of CEA was suspicious, as was immunohistochemistry of the biopsies taken, which suggested a gastrointestinal origin for the tumor. During CT scan, a tumor originating from the right ovary was seen, suggesting ovarian carcinoma. However infiltration of ovarian carcinoma in the vagina is rare.

It was unexpected that the tumor had its origin in the appendix. Appendiceal carcinoma is very rare; it has an incidence of 0.12 cases per 1,000,000 people per year [7]. Primary malignant tumors of the appendix only account for less than 0.5% of all intestinal tumors. Primary appendiceal malignancies are classified into three types: carcinoid tumors, mucinous cystadenocarcinomas and adenocarcinomas. Primary adenocarcinomas of the appendix are approximately 10 times less common than appendiceal carcinoids [8].

Mostly appendiceal carcinomas present with acute right lower abdominal pain suggestive of appendicitis. Appendiceal carcinoma can also present as a palpable abdominal mass, acute intestinal obstruction or ascites. Most appendiceal malignancies are diagnosed from histological analysis of surgically removed specimens after a simple appendectomy [8,9].

Our patient's previous hysterectomy probably allowed the tip of the appendix to move near to the vaginal vault thus causing the infiltration. Fourteen months after the initial diagnosis, our patient had recurrent disease with peritoneal carcinomatosis. The prognosis of peritoneal carcinomatosis of colorectal origin can be improved by peritonectomy followed by hyperthermic intraperitoneal chemotherapy, although this option was not considered appropriate for our patient because of her physical condition and the high morbidity and mortality risk of the procedure [10].

In one previous case report, a patient with an appendiceal carcinoma presented with post-menopausal blood loss which was caused by a metastatic tumor affecting the uterus, fallopian tubes, ovaries and peritoneal cavity [11].

## Conclusion

Post-menopausal bleeding in a patient with a history of hysterectomy is uncommon. This case highlights the need to conduct careful examination of a patient to exclude the possible non-gynecological origin of vaginal bleeding.

## Consent

Written informed consent was obtained from the patient for publication of this case report and any accompanying images. A copy of the written consent is available for review by the Editor-in-Chief of this journal.

## Competing interests

The authors declare that they have no competing interests.

## Authors' contributions

HvH and LvH were major contributors in writing the manuscript. MvD analyzed the pathology and contributed the pathology results. All authors read and approved the final manuscript.

## References

[B1] Dutch Society of Obstetrics and Gynaecology (NVOG) http://nvog-documenten.nl/index.php?pagina=/richtlijn/pagina.php&fSelectTG_62=75&fSelectedSub=62&fSelectedParent=75

[B2] Dijkwel GA van Huisseling JCM Two post-menopausal women with vaginal bleeding due to non-gynaecological malignancies Ned Tijdschr Geneeskd 2005 149 2649 2652 16358611

[B3] Medverd JR Dubinsky TJ Cost analysis model: US versus endometrial biopsy in evaluation of peri- and postmenopausal abnormal vaginal bleeding Radiology 2002 222 619 627 10.1148/radiol.2223001822 11867775

[B4] Brenner PF Differential diagnosis of abnormal uterine bleeding Am J Obstet Gynecol 1996 175 766 769 10.1016/S0002-9378(96)80082-2 8828559

[B5] Heller DS Kambham N Smith D Cracchiolo B Recurrence of gynecologic malignancy at the vaginal vault after hysterectomy Int J Gynaecol Obstet 1999 64 159 162 10.1016/S0020-7292(98)00245-8 10189025

[B6] Swan SH Intrauterine exposure to diethylstilbestrol: long-term effects in humans APMIS 2000 108 793 804 10.1111/j.1600-0463.2000.tb00001.x 11252812

[B7] McCusker ME Cote TR Clegg LX Sobin LH Primary malignant neoplasms of the appendix Cancer 2002 94 3307 3312 10.1002/cncr.10589 12115365

[B8] Lyss AP Appendiceal malignancies Semin Oncol 1988 15 129 137 3285476

[B9] Tucker ON Madhavan P Healy V Jeffers M Keane FBV Unusual presentation of an appendiceal malignancy Int Surg 2006 91 57 60 16706105

[B10] Verwaal VJ Bruin S Boot H van Slooten G van Tinteren H 8-year follow-up of randomized trial: cytoreduction and hyperthermic intraperitoneal chemotherapy versus systemic chemotherapy in patients with peritoneal carcinomatosis of colorectal cancer Ann Surg Oncol 2008 15 9 2426 2432 10.1245/s10434-008-9966-2 18521686

[B11] Alenghat E Talerman A Path FRC Adenocarcinoma of the vermiform appendix presenting as a uterine tumor Gynecol Oncol 1982 13 265 268 10.1016/0090-8258(82)90038-5 7076041

